# Monitoring Neuromuscular Performance in Military Personnel

**DOI:** 10.3390/ijerph17239147

**Published:** 2020-12-07

**Authors:** Justin J. Merrigan, Jason D. Stone, Andrew G. Thompson, W. Guy Hornsby, Joshua A. Hagen

**Affiliations:** 1 Rockefeller Neuroscience Institute, West Virginia University, Morgantown, WV 26505, USA; jason.stone1@hsc.wvu.edu (J.D.S.); andrewg.thompson@hsc.wvu.edu (A.G.T.); william.hornsby@mail.wvu.edu (W.G.H.); joshua.hagen@hsc.wvu.edu (J.A.H.); 2 College of Physical Activity and Sport Sciences, West Virginia University, Morgantown, WV 26505, USA

**Keywords:** military personnel, force plates, neuromuscular fatigue, countermovement jump, drop jump, isometric-mid-thigh pull, squat jump, tactical athletes, soldiers

## Abstract

A necessarily high standard for physical readiness in tactical environments is often accompanied by high incidences of injury due to overaccumulations of neuromuscular fatigue (NMF). To account for instances of overtraining stimulated by NMF, close monitoring of neuromuscular performance is warranted. Previously validated tests, such as the countermovement jump, are useful means for monitoring performance adaptations, resiliency to fatigue, and risk for injury. Performing such tests on force plates provides an understanding of the movement strategy used to obtain the resulting outcome (e.g., jump height). Further, force plates afford numerous objective tests that are valid and reliable for monitoring upper and lower extremity muscular strength and power (thus sensitive to NMF) with less fatiguing and safer methods than traditional one-repetition maximum assessments. Force plates provide numerous software and testing application options that can be applied to military’s training but, to be effective, requires the practitioners to have sufficient knowledge of their functions. Therefore, this review aims to explain the functions of force plate testing as well as current best practices for utilizing force plates in military settings and disseminate protocols for valid and reliable testing to collect key variables that translate to physical performance capacities.

## 1. Introduction

**Figure 1:**
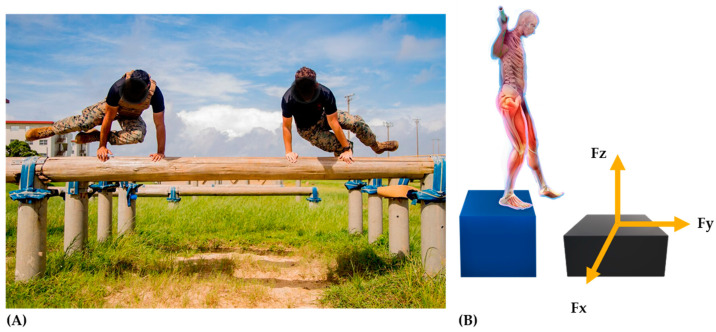
Practical training scenario of soldiers jumping over and landing from obstacles in loaded (weighted vest) and unloaded conditions (**A**). The use of drop jump testing onto force plates; (**B**) to understand the amount of forces (Fz, vertical ground reaction forces, vGRFs; Fy, saggital plane; Fx, frontal plane) occurring during landing tasks. The appearance of U.S. Department of Defense (DoD) visual information does not imply or constitute DoD endorsement.

The physical and tactical training of the military is designed to maximize operational performance capacity. The purpose of training regimens prior to mission engagement is to adequately prepare military personnel for the physical and psychological demands of their mission [1,2]. To be effective, military personnel must possess enough muscular strength, power, and endurance capabilities to complete endurance marches across rugged terrain, swiftly maneuver under fire, and engage in close quarters combat, all of which may be sustained for long durations while carrying external loads [1,2,3] (Figure 1). Although tactical training strategies are designed to produce positive longitudinal adaptations, overtraining or chronic fatigue-driven decrements in operational performance can occur [4]. Deeper insights can be achieved by objectively measuring neuromuscular performance (NP) capabilities, which encompasses monitoring: (1) physical readiness, (2) neuromuscular fatigue (NMF), (3) injury risks, and (4) adaptations to training cycles.

Objective monitoring of NMF, which may be defined as decrements in force generating capabilities, is a common practice in human performance sciences [5]. In accordance with the long lasting and widely accepted Selye’s General Adaptation Syndrome (GAS), if progressive overloading is not properly managed, military personnel can expect to reach the physiological state of overtraining, as indicated by accumulated NMF [6,7]. However, GAS suggests a minimum amount of training-induced NMF, sometimes referred to as functional overreaching, is necessary to initiate adaptations to prepare physiological systems for future high-level stressors. Yet, since there is an exceptionally high demand for physical readiness in tactical environments, military training frequently comprises bouts of high intensities and/or volumes of training in maladaptive conditions, which are often accompanied by high incidences of musculoskeletal injuries (MSKI) [8,9]. The overaccumulations of NMF, which are present in military factions all over the globe, are inadvertently costing inordinate amounts of funding and resources, annually [10]. Excessive NMF is a major contributor to overuse injuries that are rampant in military personnel [11], such as muscle strains, joint sprains, and stress fractures [11,12,13,14,15,16,17]. Consequently, mission success is directly impacted by the deleterious side effect from over accumulated NMF, such as decreased strength and power production, decreased cardiorespiratory endurance, and increased risk for MSKI [4,18]. The MSKIs subsequently result in restricted operational capacities and increased compensatory workforce strain, provided other military personnel may then be expected to account for the absence (due to injury) of others [8]. Consequently, it is imperative military groups strongly consider strategies for (1) objectively quantifying the acute effects of training loads and (2) track longitudinal responses to training stimuli to ensure positive neuromuscular performance (NP) adaptations and mitigation of injury risk [8].

Fortunately, decades of performance science research supports a variety of effective strategies for reliably and objectively assessing neuromuscular performance under virtually any circumstance (e.g., regardless of budgets, personnel resources, or time availability). One of the most common, and arguably the easiest, strategies to monitor NP in tactical populations is countermovement jump (CMJ) testing [3,19,20,21,22,23,24,25,26,27,28,29,30,31,32]. The variations of CMJ testing (e.g., tape measure, inertial measurement units, linear transducers, optical measurement systems, jump mats, and force plates) retain degrees of reliability for jump height estimates while allowing practical options for numerous occasions (e.g., widely affordable options based on budget), which explains the popularity in utilizing this testing strategy. The CMJ, a measure of lower body power output, is positively associated with occupational performances in tactical settings [25,33,34]. Further, the CMJ has been used for neuromuscular performance testing in sport and tactical environments to identify performance adaptations [23], resiliency to fatigue [35,36], and risks for injury [37]. Traditional jump testing is most simply conducted using a tape measure, vertically affixed to a wall. However, this method restricts practitioners to a single outcome measure, vertical jump height. Although this metric is generally useful, it fails to provide more valuable information on movement strategies (i.e., how the individual arrived at this outcome) and relevant weaknesses. For example, military personnel may exhibit the same jump heights before and after bouts of rigorous training, but the neuromuscular strategies used could be drastically different (e.g., countermovement depth, impulse). Thus, monitoring force-time metrics during testing may be more conducive for detecting NMF than the physical outcome (e.g., jump height) [38].

Recently, due to increased availability of both hardware and software, more sophisticated procedures including force plates have been adopted by practitioners (i.e., administrators and testers, tactical strength and conditioning facilitators). The application of NP monitoring via force plate testing presents practitioners a multitude of software and testing application options. Most solution sets provide data outputs composed of hundreds of different testing metrics, leaving it up to the practitioner to choose which metrics to monitor. This blessing and curse often leave practitioners quickly inundated with mass quantities of testing data that is poorly understood and often improperly actioned compared to common singular outputs (i.e., jump height). Therefore, it is particularly important that practitioners planning on using force plates take great care in educating themselves on the best practices of force plate testing prior to their use as monitoring tools. Under the right circumstances, force plates may be considered the most reliable method of measuring vertical jump height [39]. Additionally, due to the high sampling frequencies typically housed within force plate testing hardware, force-time data metrics are more sensitive (than jump height) to changes in the neuromuscular status of an individual, making them more effective NP monitoring strategies [40,41]. In short, reliable and quantifiable NP monitoring can be obtained using force plate testing when utilizing best practices.

Force plates provide the ability to test a large variety of neuromuscular assessments for physical capacities of strength and power. For example, isometric strength testing (e.g., isometric mid-thigh pull, squat, or bench press) may be a more appropriate means to analyze maximal force production (maximal strength) than one repetition maximum testing, as they are relatively simple to administer, time efficient, have a lower potential for injury risks, and possess high degrees of reliability under the correct testing conditions [42,43,44]. Thus, force plates provide the ability to monitor neuromuscular function more frequently and allow for testing of upper and lower body power and strength, all key components of performance monitoring in tactical settings. Therefore, this review has two purposes (1) describe how the use of force plate testing may improve physical training in the military, (2) explain the importance of using the correct applications, assessments, and metrics of interest for particular applications within tactical training of the military.

## 2. Defining Ground Reaction Forces

Biomechanics refers to the study of mechanics utilized during and resulting from body movements and is made up of kinematic and kinetic characteristics. The kinematics represent movement through positional and time data (spatiotemporal features), which in turn provides description of the movement’s position and velocity in respect to time. Kinetics refers to the interactions of forces and torques created in the body and those that may act on the body during movement (i.e., contact forces). Establishing an understanding of the forces during movement provides knowledge on how an individual achieves a desired outcome (jump height) and the effect of the forces (absorption in lower extremity joints during landing, Figure 1) on the body. Thus, by assessing applied and resultant forces during movement, analyses from force plate testing can help to explain why and how the body moves in the way that it does. Here, the end goal is to optimize movement strategies, which can translate to enhanced operational readiness via improved physical performance capacity, and reduced injury risks.

Motion results through some combination of muscle forces and external resistance forces (although it is possible that no motion occurs, and the action is isometric). It is important to differentiate force from the term torque, as torque is referring to the application of forces resulting in a rotation of a body segment. However, the focus of this review will be on the resultant vertical ground reaction forces (vGRF; i.e., forces acting on the body from the ground), which are measured in Newtons (N). Force induced movement may primarily be explained by Newton’s three Laws of Motion [45]. The first, Law of Inertia, states that an object in motion will stay in motion unless acted upon by a force. Thus, if someone is moving quickly in a sprint or falling from an obstacle, they must produce enough muscular force to overcome the inertia and controllably cease movement, making maximal strength and power valuable physical traits for military personnel. The second, Law of Acceleration, explains that the likelihood of an object to change speed or direction (acceleration) is a function of the object’s mass and force applied by it (Force = mass × acceleration). This law of motion is key for explaining the importance of force producing capabilities of tactical populations. If an individual improves leg strength and maintains body mass, they will improve their ability to accelerate and decelerate their body using their legs, which, for example, may be quantifiably observed by improved drop jump performances. The third, Law of Reaction, states that for every action there is an equal and opposite reaction, which explains the interaction between the muscle forces in the body and opposing forces of the environment. During a CMJ, the forces created by the legs against the ground will push back on the body and result in the body moving in the direction of the forces, in this case upwards. By combining these laws, it is understood that the amount of resultant forces dictates the movement characteristics that represent neuromuscular performance capabilities (i.e., greater forces created by an individual elicits a higher CMJ).

Although the amount of forces during the vertical jump may dictate the maximal height reached, there are many techniques to generate these forces that may result in the same jump height. Here, we consider the impulse (explained in detail later), which may be a result of a high amount of force over a short duration or a lower amount of force in a longer duration. Of course, vertical jump height is a valuable performance metric, but it may not be as sensitive of a metric for assessing changes over time compared to the force characteristics produced during the movement (known as the force-time curve) [35]. It is also possible to quantify the motion, known as momentum, of an individual as the product of velocity and body mass. In short, a greater impulse will yield increased momentum and if mass remains the same, velocity, and subsequently jump height, would be increased (referred to as the impulse-momentum theorem).

In order to alter the impulse during movement, one may increase their ability to produce force through training both physiologically and technically. Although anatomical structures, such as varying limb length ratios, may influence the mechanics of movement, they cannot be completely altered. However, utilizing the most efficient and safest mechanics of movement may help to improve performance and reduce the risk of injury [30]. In ergonomics, forces may be assessed to improve footwear designs or equipment load dispersion to improve the impact on forces and limb imbalances on movement quality, subsequently decreasing injury risks [46]. Since military boots may alter movement kinetics [46] it is necessary to standardize, as much as possible, the equipment used during testing. Using partial or full-kit military equipment during testing has altered movement mechanics and increased ground reaction forces and will more closely resemble that of real-world scenarios [47]. However, these controlled environments may still have limitations to the transferability to typical military movements, as fatigue, vision impairment, and distractions from secondary cognitive tasks may have further effects on landing forces [48,49,50]. Yet, as mentioned throughout this review, these force plate assessments provide valuable insight into muscular power and strength capabilities, which are closely associated to many occupational tasks within the military. Notably, force plate data are in reference to the body as a whole (i.e., center of mass) but can be broken down into right and left body segments via bipedal force plates. Thus, the use of bipedal force plates allows the ability to assess limb asymmetries during movements and provide another biomechanical metric to determine likelihood for injury and profiling of an individual. In summary, analyzing the forces of gross movement are a critical component to program effectiveness for neuromuscular performance monitoring.

## 3. Force Plate Functions

**Figure 2:**
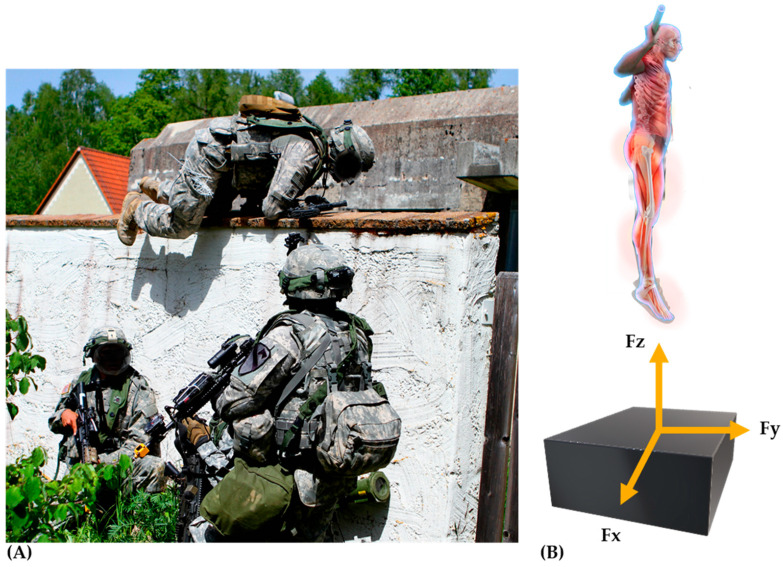
Practical operational scenario of soldiers jumping over a wall during training while under external loads (full kit) (**A**). The use of countermovement jump testing on force plates; (**B**) to (1) understand the amount of forces produced (Fz, vertical ground reaction forces, vGRFs; Fy, saggital plane; Fx, frontal plane); (2) estimate jump height, reactive strength capabilities, and power output; (3) compare performances under unloaded and loaded conditions for interpreting the ability to perform under external loads. The appearance of U.S. Department of Defense (DoD) visual information does not imply or constitute DoD endorsement.

Before selecting the test of interest, it is important to understand the basic foundational principles of force plates to improve the quality of data received. Extensive developments in force plates contributed to more sophisticated builds that include either strain gauges or piezo-electric materials, which allow higher sampling frequencies, less deformation, and more reliable data. A detailed explanation of these devices may be found elsewhere [51], but in short, piezo-electric sensors are more sensitive to detecting small rapid forces, while strain gauges are more conducive for measuring large forces particularly during longitudinal monitoring (due to the lower likelihood of signal drift). Specifically, force plates measure six variables representing reaction forces placed on the body, which equal the action forces applied by the body (i.e., Newton’s third law of motion). The ground reaction forces examined in the aforementioned tests are a cumulation of the reaction forces (*Fx*, *Fy* and *Fz*, Figure 1 and Figure 2), which are components of the four loading cells in each corner of the force plate. It is important to be aware of the direction of each axis (*Fx*, *Fy* and *Fz*) as this is subject to change across force plate manufacturers. For example, the vertical ground reaction force and focus of this review may be identified in the Y or Z axis depending on the defining source (i.e., force plate manufacturers). Additionally, it is important to consider the force plate is utilizing action-reaction principles to display force-time curves. Thus, a downward force acting on the platform is recorded as a positive force output and vice versa, making it critical to verify the direction of the output.

When considering force plate testing in NP monitoring, it is essential to identify the equipment specifications needed to ensure accurate readings. For example, the strain gauge and piezo-electric sensors may better serve different purposes (athlete fatigue monitoring and profile testing versus finger dexterity). In addition, there are certain specifications force plate signaling conditions should meet, which are explained elsewhere in more detail [52]. Ultimately, modern devices should be suitable for the purposes mentioned in this text, but practitioners should be aware of differences between devices. One concern is comparing results between different devices due to the influence of varying specifications. Additionally, to ensure reliable data, device installation should conform to manufacturer guidelines (i.e., not to place the platform on suspended floors) and practitioners should be aware potential problems may exist if these guidelines are not met. Location and use of the platforms may result in force artifacts during signal acquisition, which require post-process filtering to ensure accurate readings. For example, results will likely differ if testing takes place on a grass field compared to a level concrete floor. Although, the chief concern is ensuring identical data collection procedures across comparisons (or testing timepoints); it is also important to meet minimum specifications considering sampling rates below 300 Hz may underestimate jump height [39].

Signals from the force plate voltage sensors are recorded through analogue to digital conversion hardware linked to the collection device (e.g., computer), using a 12 or 16-bit device. Typically, the 12-bit is adequate for up to 10,000 N, within limits of most sports force measurements, while a 16-bit converter may provide excessive sensitivity. Typically, this is not a concern for most force plate practitioners, as signal processing is completed by the manufacturer’s proprietary software. Of course, there is the ability to custom write software with appropriate skills and packages, such as MatLab (MathWorks, Natick, MA, USA) or Labview (National Instruments, Austin, TX, USA). However, it is important to understand manufacturer processing methods to ensure they meet testing requirements. For example, manufacturers or custom software will utilize different filtering and processing techniques, which may under- or over-estimate the force-time curve results. It should also be noted, compared to various cutoff frequencies of filtering processes, raw vertical force data seem to produce the least amount of error [39,53]. Residual analysis via visual identification of a best fit line (that does not attenuate peak forces of various cut-off frequencies) is recommended for determining low pass filtering cut-off frequencies in custom software.

The force plate calibration process, which is often overlooked, must also be confirmed. Although relatively easy to use, force plates can be complex and require caution and attention to detail to ensure accurate data [54]. Before the platform is shipped it is typically calibrated by the manufacturer, but this does not account for individual variations of signal noise due to software processing, installation, and local environment, as well as any changes to these variables over time. Calibration processes for vertical forces are relatively simple and normally consists of placing a dead known weight on the force plate, which has been utilized by many investigators [54,55,56]. However, calibration of the horizontal forces is much more difficult to accomplish and, without verified experience, should be left to the manufacturer to reduce the risk of errors, as the magnitude and placement of the load used are critical [54,56,57]. None-the-less, calibration of the vertical force axis is easily conducted and should be incorporated into testing protocol schedules.

## 4. The Force-Time Curve Explained

**Figure 3:**
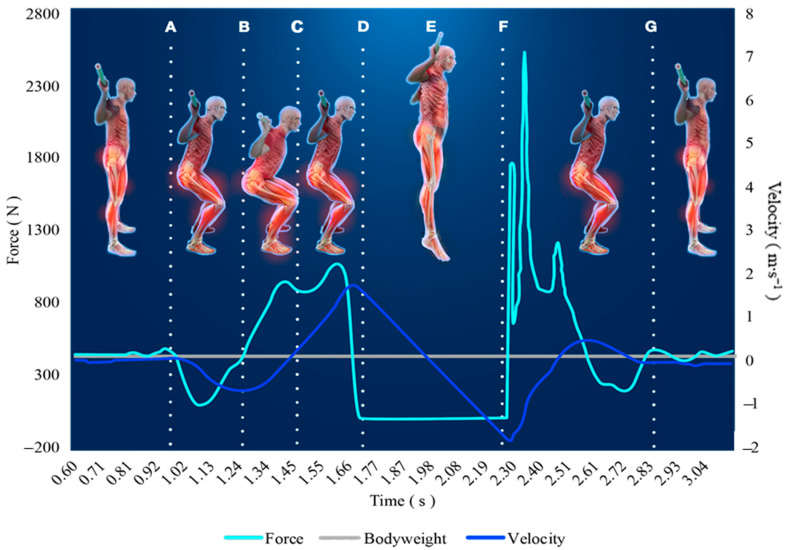
This is an example of a force-time curve from a countermovement jump (CMJ). The weighing phase occurs for at least one second prior to initializing movement (point A). The individual begins descent (i.e., eccentric action) resulting in an initial drop in the forces, known as the unweighting phase, until bodyweight is reached (point B). The individual must decelerate through the remainder of the eccentric phase until velocity reaches zero (point C), known as the braking phase. The individual then explodes upwards until take-off (point D), reaching maximal jump height approximately half way through the flight phase (point E), and eventually landing (point F). The landing phase then begins until a stabilization period is reached (point G).

**Figure 4:**
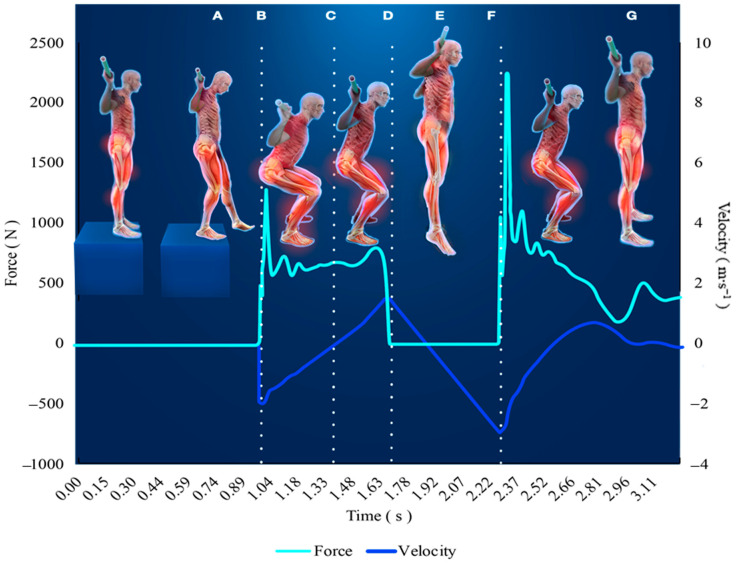
This is an example the force-time curve from a drop jump (DJ). The weighing phase still occurs for at least one second, but does not include capturing the individuals bodyweight. The individual begins the drop jump by stepping off, not jumping from or stepping down from, a standard height (point A) until coming into first contact with the ground (point B) and beginning the eccentric (i.e., braking) portion of the initial landing phase until velocity reaches zero (point C). As quickly as possible, the individual then explodes upwards until take-off (point D), reaching maximal jump height approximately half way through the flight phase (point E), and eventually landing (point F). The landing phase begins until a stabilization period is eventually reached (point G).

**Figure 5:**
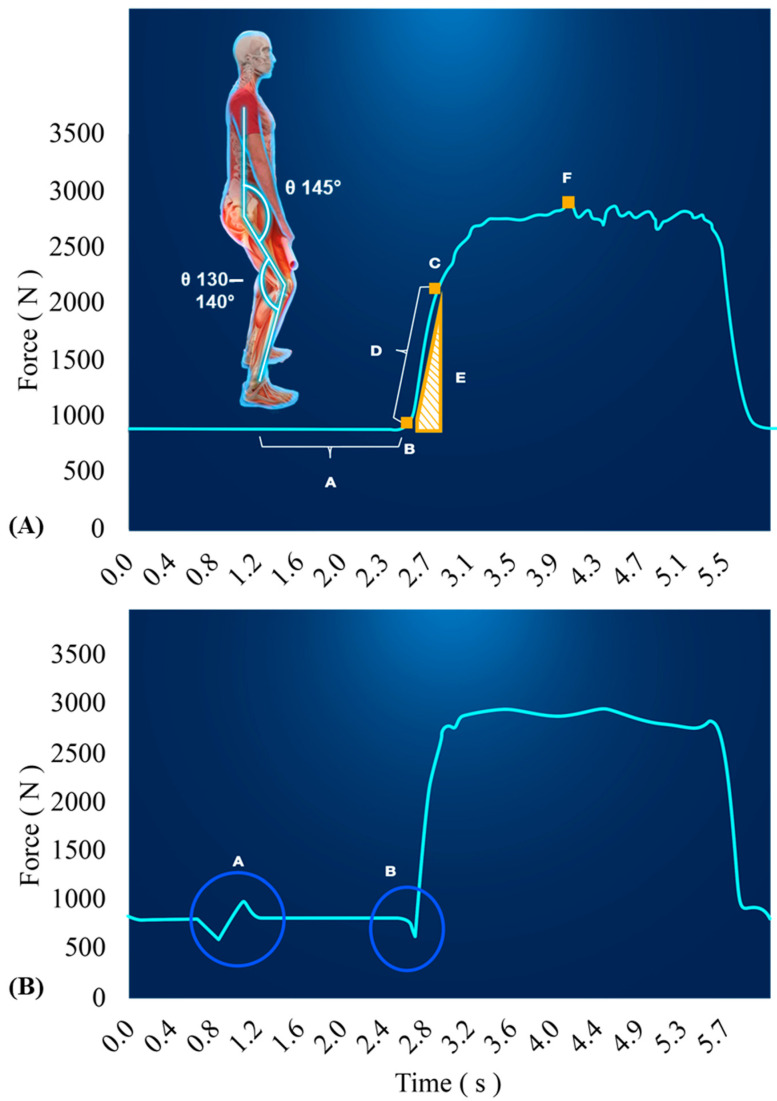
A correct example of a force-time curve from an isometric mid-thigh pull (IMTP) is presented in (**A**) including a one second weighing phase (point A). Example variables of interest may exist in time epochs from 0 (initiation of movement, (point B) to 250 milliseconds (point C), such as the slope of the force-time curve (point D) or the area under the force-time curve, known as impulse (point E). Additionally, peak force (point F) is calculated and often most reliable. In (**B)**, common errors in assessing the IMTP are presented, such as too much movement during the weighting phase (point A) and a countermovement prior to pulling on the bar (point B).

The vGRFs are sampled over time creating what is known as a force-time curve. Force-time curves of various exercises have been used to evaluate and monitor skeletal muscle function [36,58,59]. When monitoring NP using force-time curves obtained by force plates, the extent of variables provided is quite remarkable. It is most common to quantify mean and peak forces and rate of force development (RFD), also known as maximal force generating capacities and the slope of the force-time curve, to assess various performance qualities [60]. Stereotypical differences in movement patterns permit force time curves to be summarized in a few shapes, such as: dynamic movements starting on the force plate (Figure 3; e.g., CMJ, SJ, Clapping push-up), dynamic movements starting off the force plate (Figure 4; e.g., drop jump or push-up), isometric strength tests (Figure 5; IMTP, isometric bench). None-the-less, there are key considerations specific to the equipment, movement, and data for ensuring the force-time data are valid and reliable. Several of these necessary considerations are discussed in this section and summarized in Table 1.

### 4.1. Importance of Measuring Body Weight

For a detailed example of walking through all the phases of a movement, readers are directed to a well outlined article, by McMahon et al., covering the phases of a CMJ [61]. Prior to determining the key phases in any movement, it is crucial to meticulously measure the individual’s bodyweight, as this will be utilized to identify the onset of movement and derive acceleration data. It is encouraged that bodyweight be calculated by controlling for the inherent noise in the force plate by subtracting the peak residual forces during the flight phase from the original bodyweight calculation of average force recorded during the weighing phase [61]. However, this method will not account for the noise imposed by an individual’s movement during the weighing phase (Figure 5B). Therefore, it is absolutely critical to testing data quality that the individual being tested stands as upright and still as possible during this phase to ensure accurate bodyweight measurements, as a 0.5% error in bodyweight can induce remarkable errors in jump height calculations and subsequent inferences from data analysis [62].

### 4.2. Identifying the Onset of Movement or Distinction of Phases

Next, the onset of a movement is generally identified when forces fall either above or below a certain threshold. In the case of a CMJ, the most accurate way to identify the onset of movement is with a threshold set at five standard deviations of bodyweight [63]. In this case, the error from an individual’s movements will increase the standard deviation of bodyweight measurement, incorrectly shifting the initiation of movement further right on the force-time curve, thus occurring after movement commencement has already taken place. This may not have as much of an effect on forward dynamics (estimating velocity and positional data), but would likely alter any time variables, such as time to peak force, time to take-off, and associated variables, such as RFD, impulse, and reactive strength indexes. Regardless of movement onset calculation method, it is critical to utilize consistent measurement techniques and thresholds to compare within and between individuals. Although some have used absolute values, such as when the vGRFs reach above 40 N, 5 standard deviations above the mean of the weighting phase is recommended for identifying the onset of movement [53]. This will account for variability during the weighting phase and reduce the influence of signal noise on the calculations. Thus, it is important all force plate testing includes at least one second of a quiet, still weighing phase [64]. For the isometric tests, it is important standing time does not include any tension or countermovement prior to initiating the pull or squat. In other words, the individual should not pull on the bar before being instructed to or dip down (at the hip and/or knees) before pulling upwards. Otherwise, the reliability of the data will be compromised, specifically the identification of the movement onset.

In the CMJ, other phases such as the cessation of the unweighing phase, are also identified using bodyweight thresholds, so errors in bodyweight measures will result in continued error through the force-time curve analysis. However, there are other examples where “bodyweight” is not measured, but the same principles are applied. For example, the flight phase of the CMJ is considered as the time between leaving and returning to the force plate. The same threshold of 5 standard deviations is applied to the forces during a 300 milliseconds period of the flight phase to identify take-off and landing [53]. Likewise, during a drop jump, the individual begins off the force plate, and the reading during the weighing phase would be around 0 N. To account for the noise in the force plate signal, a threshold of 5 SD during one second of that phase should be used to identify landing from the desired box height.

The landing phase is often of interest as it provides insight into how well an individual may absorb the forces during landing. Main factors in landing force impulses are the mass of the individual and the landing velocity. Thus, under loaded conditions, individuals are tasked with altering mechanics in an attempt to absorb the forces and reduce the increase in impact from external load. Similarly, landing velocity will be dictated by the height one is landing from. In the vertical jump, this means higher jumps coincide with faster landing velocities and require larger net impulses to come to a complete stop [65,66]. To absorb the landing, it is often instructed that the individual applies smaller forces over a longer duration by flexing more at the hips, knees, and ankles. Otherwise, the individual will land “hard” and the forces will be greater over a shorter period of time. Therefore, it is important to note that landing forces from a vertical jump will significantly vary with jumping height. Thus, a more appropriate test for assessing the ability to absorb forces during landing tasks would be a drop jump since the height of the landing is standardized.

### 4.3. Integrating the Force-Time Curve

Care must also be taken when utilizing force plates to estimate the movement of an individual’s center of mass (i.e., jump height). Considering force is a product of mass and acceleration, it is possible to compute acceleration data from the force plate and integrate acceleration to compute velocity and positional data about the center of mass. Yet, these integration techniques require certain precautions outlined by Vanrenterghem et al. [62]. Although integration techniques have improved and are considered reliable, more deviation from the raw force data may result in larger degrees of error. However, this is not meant to discourage the use of velocity or positional metrics from force plates. Instead, it is to stress the importance of using the same techniques to achieve the data for comparisons, because differing techniques of integration and devices may not be appropriate if the same sampling rates are not used. At the very least, integrating the force-time curve to simultaneously evaluate velocity- and positional-time curves may allow more support for correctly identifying phases of the movement. Methods to conduct these procedures can be found elsewhere [58].

As seen in Figure 3, the unweighting phase coincides with an initial dip of the positional curve and the fastest negative velocity, which can be used to confirm accurate phase identification points. The same can be completed for dynamic movements where the individual begins off the force plate (Figure 2), but the integration process is altered. When the individual is standing on the force plate their velocity is 0 m∙s^−1^, until they initiate movement, but the velocity of the individual during a drop jump will be dictated by the height of the drop. Thus, standard physics calculations, using the acceleration of gravity and the set drop height, must be used to estimate the initial velocity upon impact prior to integrating the force time curve. During CMJs and drop jumps, a faster velocity and greater net impulse during the unweighing phase or initial impact requires a greater impulse to reduce the velocity to zero and begin the propulsion phase. This is why it is important to provide consistent instructions for each performance, as the same impulse can be achieved with differing mechanics (i.e., large force in less time or small force over more time). For example, if the individual is instructed to perform the jump as quickly as possible, duration needs to be reduced to slow movement velocity to zero quickly, but this may also result in less forces and a lower resultant jump height.

Further, it is not recommended to use integration to identify the peak center of mass height as maximal jump height. The recommended criterion method for estimating jump height is through the take-off velocity method, where jump height is equal to half of takeoff velocity squared divided by gravitational acceleration (½ × (takeoff velocity^2^/ 9.81 m∙s^−2^) [64]. Although the error from incorrectly identifying critical timepoints may be reduced by using adequate sampling rates and integration frequencies [39], calculating jump height by flight time rather than the impulse momentum relationship method may also be subject to more error. This process (flight time derivations of jump height) assumes peak jump height occurs halfway through flight, and is subject to human error if flexion at the ankles, knees, or hips occurs before landing and incorrectly alters flight time [67]. As a result, prior investigators have found the flight time method to result in overestimations of jump heights by as much as a few centimeters, due to disagreements between take-off and landing heights [64].

## 5. The Force-Time Curve Considerations

Selecting the right variables form the force-time curve is of upmost importance. Typically, the forces, as either mean, net, or peak forces, and duration of movement phases are of interest. Additionally, some variables may be derived directly from this force-time curve, such as impulse and RFD. The impulse of a movement refers to the area under the curve and is now typically calculated with integration techniques using the trapezium rule [39,64,68]. This variable is most often useful when a movement is broken down into phases, such as the braking and propulsive phases of a CMJ or landing phase of a drop jump. As previously mentioned, this may help explain different techniques for obtaining a similar outcome of maximal jump height, for example. The RFD is typically of interest to exercise biomechanists as many sport tasks occur in very small timeframes which require forces to be developed rapidly. Conversely, the rate of loading during the drop jump landing may be of interest and pertains to an individual’s ability to absorb forces during rapid movements, such as landing from a vehicle or obstacle. In either case, there are numerous ways to calculate RFD. One method is calculating the average slope between two arbitrary time points, be it a percentage of peak force (PF) or time bands from initiation of movement (i.e., 0–200 milliseconds). Another method is differentiating the force-time curve and computing an instantaneous value with the highest rate of force development between two time points along the force-time curve. Some have found RFD to be more sensitive to changes in NP than measures such as PF during an IMTP [69,70]; however, variables are typically more trustworthy and sensitive and to changes over time or comparisons between individuals when the metric is reliable.

### 5.1. Selecting the Right Variables

The number of variables that may be analyzed from a force-time curve is quite spectacular and, as a result, many manufacturers will provide virtually all of them, which most times is more than necessary. Unfortunately, this can easily lead to confusion for both the practitioner and tactical personnel and lead to poor understanding and compliance. Fortunately, there are a few key strategies one may use to help guide them to selecting the best variables of interest for a given test. In general, the variables selected should be easy to digest and independently informative. In other words, the variables have face validity (are a depiction of what is measured by the eye) and each tell a different story with minimum overlap among them. A good example of this is peak velocity (which typically occurs near take off) and jump height during a CMJ. Typically, jump height is calculated using take off velocity, which means the variables are highly correlated, and therefore tell the same story. However, it is easier for someone to comprehend what jump height means compared to peak vertical velocity. There is an added bonus to analyzing jump height, as the individual being tested will aim to achieve the highest jump possible and ensures that all the data obtained is from maximal effort attempts. This is a critical component to testing since a lack of effort on a given test will significantly disrupt efforts to monitor NMF and NP adaptations. This is a point of emphasis for both the practitioner administering the test and the tactical personnel performing the test: maximal effort must be ensured by all concerning parties prior to testing to ensure the data is in fact reliable and valid. The maximal height achieved during a jump is also a metric that may be easily calculated from other tools such as jump mats, vertecs, or a simple measuring tape on the wall, which is important if force plates are not available during certain times. However, as mentioned previously, these tools will not give insight into how that jump height was achieved.

Additionally, it is important to remove unreliable variables from the analysis that are subject to error. This is an easy way to reduce the numerous variables from your force plate data outputs while also ensuring the metrics being monitored are sensitive to change (fatigue or adaptation), which will make monitoring NP much more effective. The next factor to consider are the variables that directly influence the CMJ performance and neuromuscular function, which as previously mentioned will be the forces applied to the ground and all of its components. In other words, consider the peak or mean forces, the net forces, or the impulse during the entire movement or select phases of the movement. Further, the strategy one uses to achieve these forces may be useful to track. For example, the time taken to deliver the net mean forces during the braking phase of a CMJ provides insight on how long it took to descend before initiating the ascent and take off to perform the jump (longer eccentric duration that may be suggestive of NMF). From the force-time curve, impulse is calculated and is the driving factor of the movement. However, the same impulse may be achieved by having more mean force spanning less time (ideal; more explosive) or less mean force across more time. Therefore, net force, duration, and impulse are typically the most influential factors to jump performance and its associations with NP.

### 5.2. Reliable Metrics and Reliability Testing

No matter the force plate test, the key to obtaining reliable data is to have each test be performed under the same conditions across time points. Any lack of consistency in testing procedures increases the likelihood of Type I (false positive) and Type II (false negative) errors, which further lends itself to poor neuromuscular performance monitoring because of increased difficulty interpreting intraindividual changes over time or interindividual comparisons. To determine reliability, often, the intraclass correlation coefficient (ICC) is used as the degree of consistency and agreement between two sets of data; however, it does not account for systematic errors that may result in correlated data sets to be non-repeatable. Thus, it is important that the coefficient of variation (CV) is also reported to provide the typical measurement error [71]. For force-time characteristics there is no minimal acceptable thresholds for these values, but acceptable reliability is typically considered as an ICC ≥ 0.80 and a CV ≤ 10% [72]. More robust procedures would also include the confidence intervals (95% is most common) around the reliability measures to indicate the range of reliability found during testing, since the limits above and below the average value may not be acceptable.

For RFD, time bands of 0–50, 0–150, and 0–250 milliseconds are more reliable than the peak RFD or average RFD. The time bands are calculated as the change in forces divided by the time (50–250 milliseconds), while the peak RFD is the highest rate of force development occurring at a sampling window. Average RFD is calculated as the peak force divided by the time to achieve peak force. The issues lie in the fact that the time bands are so small for the highest RFD that it is likely to vary quite a bit and/or be attributed to artifact. However, if the slope of the force-time curve is steeper in the beginning of the movement, more total net forces are achieved in that duration and thus, the total impulse would also be increased. Furthermore, impulse is a more reliable measure and assessing various time bands from 0–100, 0–200, and 0–300 milliseconds are recommended over peak or average RFD over the entire IMTP effort. There have been other calculations for isometric testing, such as index of explosiveness, reactivity coefficient, S-gradient, A gradient, but have been found to be particularly unreliable [42]. Consequently, for isometric strength assessments it is recommended to analyze the peak force of the entire movement and impulse at epochs from 0–100 to 0–300 milliseconds, as these are the most reliable measures and most likely to accurately detect change in strength metrics over time.

### 5.3. Conducting Multiple Versus Single Trials

Further, it is recommended that multiple trials of each test be recorded if time permits. Although a singular trial may be sufficient for monitoring NMF if multiple trials are not feasible [23], an individual’s performance may increase from trial 1 to 2, as the musculature potentiates from the previous trial. Yet, the maximal effort of an individual is often difficult to determine and if maximal effort is not always provided the recording and analysis of the maximal effort strength and power tests over time may not be accurate. For this reason, others have found that the average values are more appropriate for monitoring NMF and performance adaptations [73], while others have found similar results between the highest and average jump height performance [74]. From a statistical standpoint, the average of scores is also more likely to be the truer performance result, and when monitoring NP it is essential to obtain “true” scores to accurately detect change [36,72,75]. Thus, multiple trials allow for in house testing of reliability checks, as well as average trial results which typically are more sensitive to changes in neuromuscular performances [36]. Lastly, if a single trial is used, it is strongly encouraged that standardized and supervised warm-up procedures are used including multiple jump trials prior to collecting data on a single trial to improve the test’s validity.

## 6. Standardized and Validated Tests for Force Plates

There are many existing standardized and validated tests that are useful to monitor in tactical settings, such as various plyometric and isometric testing protocols. Although each test has some similar data collection and analyses, as well as variables of interest, there are distinct differences to consider. The remainder of this section will describe and provide rationale and guidelines for conducting each test with the tactical personnel in mind. A summary list of metrics and their definition is presented for jump testing in Table 2. A short summary is included, in Table 3, at the end of the section to outline several neuromuscular performance tests, reliable variables for each test, and their relevance to tactical populations.

### 6.1. Lower Body Power Testing

#### 6.1.1. Countermovement or Squat Jump (CMJ or SJ)

The CMJ is one of the most common and easiest tests to administer, is thoroughly examined, and as a result, is a longstanding strategy for monitoring the neuromuscular status of athletes in sport and tactical settings [19,20,21,22,23]. Moreover, previous research has demonstrated that CMJ height is related with tactical occupational performances [25,33]. Many kinetic and kinematic variables may be assessed from the CMJ’s force-time curve (Figure 3; Table 2), but some variables may be more reliable during testing and, thus, more sensitive to change in the neuromuscular status of an individual [40,41]. There are also a variety of ways to test vertical jump performances. For example, a countermovement may or may not be warranted, the latter typically referred to as a squat jump (SJ), in an attempt to isolate the stretch-shortening cycle and its influence (or lack thereof) on force generating capacities. Within each jump type, there may also be unloaded or loaded conditions to elucidate the influence of external loads on neuromuscular performance. The external load can be placed on the individual via a weighted vest, barbell, or dumbbells, but it is important to be consistent with the amount of load used and its placement. Selecting which type of test to perform should be dictated by the specific nature of the occupation, military occupational specialty (MOS), and task performance demands. For example, in athletic populations, who seldom or never train under external loads, unloaded CMJs are more commonly used as a monitoring tool [35]. In tactical populations, individuals are far more likely to be under some sort of external load, be it full kit or only plate carriers. Consequently, loaded assessments may be more sensitive to changes in NP under conditions possessing greater ecological validity. Therefore, they may provide an especially valuable assessment strategy to understand an individual’s loaded performance ability, but also closely monitor whether NMF is accumulating. Likewise, the type of fatiguing scenarios may stimulate different responses specific to the various jumping protocols, be it static SJs, unloaded or loaded CMJs, or drop jumps [35].

Meanwhile, the difference between CMJs and SJs is the utilization of the full stretch-shortening cycle. During the CMJ, as indicated in Figure 3, there is an eccentric phase followed by a concentric phase, which are separated by a preferably short amortization phase (i.e., the individual squats down and jumps up as quickly as possible). However, a SJ is only concerned with concentric performance (upward movement forces) and begins from a set starting depth (knee flexion measured at 90°). Thus, the SJ has the same force-time curve as a CMJ without the initial eccentric phases, which is less often used in practical scenarios [64]. Comparatively, the CMJ may be more applicable to most human locomotion since concentric phases are typically preceded by eccentric phases, such as in running or breaching a barrier. However, monitoring both may better afford assessing utilization of the stretch shortening cycle, as the CMJ height should be higher than the SJ. One SJ benefit may be the ability to precisely control starting knee angle, while the CMJ may provide improved determination of explosive capabilities through more advanced biomechanical calculations, such as the modified reactive strength index (Table 2). It should be noted that discrepancies exist on whether the SJ or CMJ is more sensitive to changes due to NMF [76,77]. Furthermore, jump height may be a valuable indicator of chronic NP training adaptations, but force-time metrics and countermovement dip displacement may be more sensitive to NMF than jump height alone [35,36,77].

**Table 2:** Definitions of Force-Time Metrics and Related Tests.

Metric	Definition	CMJ	SJ	DJ	Depth Drop
**Performance Metrics**					
Jump Height	Greatest center of mass vertical displacement during flight (cm). Calculated using flight time or impulse-momentum theorum.	Y	Y	Y	
RSI	Ratio between jump height (or flight time) and initial ground contact time (arbitrary units).			Y	
RSImod	Ratio between jump height and time to take-off [78].	Y			
Time to Stabilization	Time from the landing point to the period of stabilization (vertical forces reach and stay within 5.0% or 5 SD of body weight for 1-second) (s).				Y
**Explanatory Force-Time Curve Metrics**					
Contact Time	Duration from initial ground contact to take-off (s).			Y	
Countermovement Depth	Maximal vertical center of mass displacement during initial ground contact (cm).	Y	Y	Y	Y
Braking Duration	Duration of the braking phase (s).	Y		Y	
Braking Mean Force	Mean force of the braking phase (N)	Y		Y	
Braking Impulse	Area under the braking phase of the net force-time curve (N×).	Y		Y	
Propulsive Duration:	Duration of the propulsive phase (s).	Y	Y	Y	
Propulsive Mean Force	Mean force of the propulsive phase (N)	Y		Y	
Propulsive Impulse	Area under the propulsive phase of the net force-time curve (N×s).	Y	Y	Y	
Mean Propulsive Power	Propulsive phase’s average power output (W).	Y	Y	Y	
Braking Velocity	Braking phase’s mean or peak (m·s^−1^)			Y	Y
Peak Landing Force	Peak force occurred during the landing phase (N).			Y	Y
Peak Landing Force Timing	Time of peak landing force relative contact time (s).			Y	Y
**Metrics to Use with Caution**					
Rate of Force Development	The change in force over time (i.e., force-time curve slope). Using time epochs (i.e., 0–250 milliseconds) are preferred, as well as internal, routine reliability assessments.				
Propulsive Phase or Total Peak Power	Amount of work performed over time. With high sampling rates (i.e., 1000 Hz), peak power describes a very short time period (i.e., 1 millisecond) and may be variable (sensitive to artifact).				

Performance metrics, the main outcome variables; Explanatory Force-time Curve Metrics, can be used to explain how individuals arrived at performance outcomes and may be sensitive to fatigue; Metrics to Use with Caution, metrics that may have low reliability or do not add further pertinent information. Braking and propulsive phases may also be referred to as eccentric and concentric phases, respectively. CMJ, countermovement jump; SJ, squat jump; DJ, drop jump.

Further, CMJ testing may be conducted with multiple continuous jumps for a given duration (i.e., 15–60 s). Prior research has found repeated jumping for 30–60 s can be used as a sensitive measure of assessing anaerobic power or fatigue patterns during strenuous tasks lasting 60 s [79,80]. Jump testing for shorter durations of 15 s may also be used to determine levels of NMF following strenuous training bouts [81]. Yet, the reactive strength index from continuous jumping assessments should not be used interchangeably with traditional CMJ or drop jump performances [82].

#### 6.1.2. Drop Jump/Depth Jump/Depth Drop

Although the CMJ may be a good indicator of lower extremity power output, the drop jump or depth jump may provide additional information on how well an individual can absorb forces (e.g., landing from a relatively high terrain or vehicle). Although there is only a miniscule difference in provided instruction, the differences between test types is important to note [83]. The drop jump instructions include cues to land and explode up with minimal ground contact, which is slightly different than a depth jump where the goal and instruction are directed to achieve maximal power and jump height at whatever means necessary. This is important to decide up front and keep consistent the instruction, as outcome variables will vary from different intent of the movement [83,84]. Specifically, the drop jump will typically be performed more explosively and result in greater reactive strength indexes, while the depth jump would result in greater jump heights and power outputs [83]. Thus, as the intent of the movement will dictate the performance outcomes and should be consistent. Otherwise, following the initial drop from the box, the same procedures are followed for drop and depth jumps as for CMJs. Force-time curve analysis will be slightly different when performers do not begin on the force plates (Figure 4); thus, threshold calculations for identifying key timepoints (i.e., onset of movement) is slightly different in that they are no longer based on body weight. Additionally, the eccentric (or braking) phase of the drop jump carries greater significance for indicating an individual’s ability and strategy to absorb forces and decelerate the body.

Conversely, if the focus is solely on assessing the ability to decelerate the body to a stable balance, the depth drop should be used. During the depth drop, the individual drops from a standardized height and lands with the intent of standing upright and coming to a still position as quickly as possible. However, it is unlikely that no movement will be required immediately following a landing scenario, and thus, the drop and depth jumps are more ecologically valid tests than the depth drop. Although landing metrics may also be recorded from a CMJ, landing forces will be influenced by differences in jump heights between trials, whereas the drop jumps entail dropping from a standardized box height. Thus, force-time metrics from the landing phase are of greater interest from drop jump performances (Table 2). Likewise, braking phase metrics are not outputted from the squat jump, as the movement is only comprised of a concentric action. In conclusion, metrics of interest are determined by the dynamic test selected and are outlined in Table 2.

### 6.2. Lower-Body Strength Testing: Isometric Mid-Thigh Pull and Squat

The enhancement of maximal force production (i.e., peak force) and ability to generate force rapidly (i.e., RFD) is instrumental to NP [85], making strength and power testing a popular means to monitor training program adaptations. However, isometric strength testing, as either an isometric mid-thigh pull (IMTP) or an isometric squat, may be a more appropriate means to analyze maximal force production (maximal strength). Reason being, isometric tests are relatively simple to administer and time efficient, reduce the risk of injury in comparison to one-repetition maximum testing, and are very reliable under the correct and consistent conditions [42,43,44]. Additionally, even though the IMTP is isometric by nature (it does not involve physical movement or displacement of body segments), measures from the IMTP correlate to performance in dynamic movements of powerlifting [86], weightlifting [87], sprinting [88], and jumping [89]. Most importantly, the IMTP and isometric squat do not require the extensive familiarization periods that may be associated with a traditional one-repetition maximal test, which requires specific skill development in the weight room (e.g., becoming highly proficient in the back squat, bench press, deadlift, etc.). Additionally, isometric testing with force plates is highly reliable under the right conditions, and likely more advantageous for determining changes in strength over time [90]. Reliability concerns need to also be addressed for isometric testing. Changing the position of an individual’s knee and/or hip angle(s) may vary the reliability and validity for force-time curve metrics of interest [43]. Importantly, more reliable metrics, such as peak force, may be useful for testing maximal strength changes over time, but are not as sensitive to NMF related changes in performance (compared with RFD metrics) [69,70]. Therefore, these isometric tests may be useful for monitoring neuromuscular performances over time, but caution and care should be taken to ensure data validity and reliability.

To start, the IMTP and squat are performed with the individual standing on the middle of a force plate using a testing system designed to fixate a bar at any desired height above the platform. The bar should be completely immoveable to reduce any “slack” and ensure accurate readings from an optimal signal to noise ratio. Another necessary step to reduce confounding variables, such as grip strength, from IMTP is to strap the individual’s hands to the bar using wrist straps and athletic tape after positioning. Typically, IMTP bar height is set at 50% of the thigh length (measured between the greater trochanter and lateral epicondyle of the knee), while the isometric squat setup positions the bar on the upper trapezius. Additionally, prior to testing, the individual’s hip and knee angles need to be measured with a goniometer. Previous research concludes the IMTP’s most reliable angles are 130–140° and 145° for the knee and hip angles, respectively (Figure 5A), which corresponds with the power position of the clean and provides the greatest peak forces [42,43]. Unfortunately, less literature exists for the isometric squat test [42], which may be due to the undue spinal stress. However, the peak force and impulse (0–250 milliseconds) during isometric squat performed at 90° and 120° knee angles are reliable metrics, related to back squat 1-RM performances, and applicable to monitor training-induced changes in strength and explosiveness [91].

Nonetheless, standardization of the knee and hip angles, foot placement, and biomechanical calculations is necessary across all testing sessions for within and between individual comparisons. For example, including body mass (zeroing the force plate while individual is standing on it) will result in much smaller forces since the weight of the individual will be removed from the force-time curve. However, there is no acceleration of body mass during the movement, so net forces excluding body mass may be recommended. Yet, there are no differences in the relationship between dynamic actions and the IMTP when body weight is and is not offset [87]. Thus, the most important considerations are to ensure consistency in data collection techniques and avoid making comparisons between net and absolute force-time values. Further, scaling the forces, which may be as simple as dividing by body mass, is an effective way to account for body mass changes over time and differences between individuals [92]. Therefore, the use of scaling allows for appropriate comparisons within an individual over time and between individuals where body mass may differ. Lastly, two important coaching cues are: (1) stand as still as possible without any tension on the bar to calculate an accurate bodyweight and (2) pull the immovable bar as hard and as fast as possible and continue pulling for the set duration (typically 3–5 s or until the practitioner verbalizes to cease effort). When attempting to achieve both peak forces and explosiveness, the latter cue helps to ensure the intention of the movement elicits maximal effort thereby confirming that the isometric data were collected reliably and is generally recommended.

### 6.3. Upper Body Isometric Strength Testing

Isometric strength testing may also be applied to the upper body in assessments for both the push [93,94] and pull [95] musculatures. All the same rules apply for upper body assessments: how the testing is conducted, the instructions provided, and analysis of the force-time curve. However, one distinct difference to consider is a bench apparatus must be placed on the force plate for the individual to lay on. Importantly, the force plate zero should include the bench and individual, prior to initiating movement. Further, the grip width needs to be standardized, along with the location of the bar in relation to the individual’s chest (i.e., aligned with the xyphoid process).

Although relatively less research exists pertaining to the upper body strength assessments, the isometric bench press and pull are considered reliable with necessary precautions similar to the lower body isometric tests. For the bench press, elbow angles of 60, 90, 120, or 150° may result in reliable peak forces, but not RFD. Some have found 120 and 150° to be slightly more reliable elbow angles [94], but others have found 90° to be just as reliable as 120°, while possessing more relevance to dynamic one repetition maximum performances [93]. Similar elbow angles may be used for an isometric prone pull test where peak force and RFD have been reliable and related to dynamic 1RM performances at 90° and 120° elbow angles [95]. Indeed, force plates offer numerous, flexible, reliable testing options in terms of assessing muscular strength. By utilizing isometric push and pull assessments one may also identify the push to pull ratio of the upper extremities, a relevant assessment for tactical populations assessing shoulder joint injury risks [96].

### 6.4. Upper Body Plyometrics

Upper extremity explosive ability is a highly desirable characteristic to optimize performance capability of tactical skills, such as hand-to-hand combat, maneuvering around, through, or over obstacles, and handling equipment. Although, most explosive power assessment monitoring and validation tests have been conducted with lower body assessments, there is also promise for upper body plyometric testing [97,98,99]. Since upper body muscular power is of importance, identifying methods to assess upper body power and infer performance potential or talent should be beneficial to monitoring program effects (NMF) and NP development. Tests may range from field-based assessments of medicine ball throw distances to measuring explosive bench throw loads in the weight room or plyometric push up assessments for time or with force plates [97,98,99,100]. Unfortunately, there is limited extant literature investigating the force-time characteristics of plyometric pushups; although, studies have indicated an acceptable reliability of force metrics within various plyometric push-ups assessments (ICC = 0.80–0.98) [97,99,101]. However, the same efforts must be taken to ensure reliable data collection, particularly familiarization sessions as this movement is less common than a vertical jump. None-the-less, it may be assumed that the reported force-time metrics of the countermovement plyometric push-ups be similar to those from the CMJ and alike variations (loaded versus unloaded).

For example, the findings that increased flexion across lower extremity joints result in absorption of vGRFs over a greater length of time and reduced peak forces during lower body plyometrics [102] can also be applied to upper extremity plyometrics [103]. Likewise, the forces that occur during upper body plyometrics are dependent on the type of test that is conducted [99]. Since upper body movements, such as striking in hand to hand combat, may occur in short durations, future research is encouraged to evaluate short time epochs (i.e., 0–50 milliseconds), including their reliability and relation to performances. Relatedly, boxing research has investigated the efficacy of evaluating force-time curves of punching variations [104]. However, research is limited and this testing variation requires mounting the force plate to the wall in a very particular manner. Thus, we include it as an option, but suggest caution and direct readers to previous publications for more details on these assessments and their practical implications [104,105].

### 6.5. Limb Assymetries

As previously mentioned, the use of bipedal force plates allows the ability to individually assess the forces produced and absorbed by right and left limbs. In fact, vertical ground reaction force asymmetries during a stop-jump task were predictive of forces at the knee joint and thus may be useful for understanding injury risk [106]. However, asymmetries should not be used as a predictor for injury or performance capabilities as there is no clear value to determine asymmetries that are advantageous or detrimental. Many factors will result in varying inter-individual baseline asymmetries such as; chronic responses from prior injuries [107], occupational specializations, anthropometrics (e.g., limb length differences), and physiology (e.g., muscle mass differences) [108]. Thus, it is necessary to compare individuals to those within the same population. If these data or values do not exist for the population of interest, then monitoring asymmetries within individuals is a helpful alternative for understanding their responses to injuries or neuromuscular training [109].

As with aforementioned performance and fatigue assessments, the ability to monitor asymmetries will rely on accurate baseline measurements. To start, identifying which tests are more sensitive to detecting asymmetries is important. For example, single leg jumping tasks are considered sensitive to detecting asymmetries between lower limbs [110], but asymmetries in bilateral jumping tasks may be lower and more difficult to detect. Furthermore, if the individual is not facing the correct direction on the force plate the orientation of left and right limbs will be incorrect as well as subsequent calculations. Additionally, there may be a learning curve to performing these tasks, especially single leg jumping tasks. Thus, consistency between trials is important to consider. In some cases the individual may show a wide range of asymmetries with some trials favoring the right, while others favoring the left side. However, the average asymmetry across all trials may be closer to 0% and thus not concerning as the individual is capable of spreading the workload across limbs. On the other hand, a practitioner should be more alarmed if an individual consistently displays a large asymmetry across several metrics and trials. Moreover, to truly understand asymmetries, analyses of metrics from various phases of a movement (i.e., concentric and eccentric actions of the CMJ), as well as various movements, are necessary. For example, an individual may be more protective of rapid or heavily loaded movements, which will be shown more for certain metrics or certain tasks.

### 6.6. Balance Assessments

Although outside of the main scope of this manuscript (being the analysis of vertical ground reaction forces), force plates can be used to assess static and dynamic balance capabilities. For example, time to stabilization from the vertical ground reaction force-time curve of a depth drop may be indicative of the ability to stabilize the body in a dynamic scenario (Table 2). However, since time to stabilization may be influenced by dropping height it should only be analyzed when dropping from a known height, as analyzing time to stabilization following a landing from a vertical jump is unreliable [78,111]. In addition to vertical ground reaction forces, analyzing anterior-posterior and medial-lateral deviation (i.e., center of pressure) and time to stabilization may be helpful for indicating responses to injuries [112]. Yet, there may be more steps to normalize for testing as the individual’s limb length may influence the TTS response and should be taken into account [113]. In addition to dynamic balance, static balance (i.e., postural sway, Sensory Organization Test) may be useful for detecting balance impairments following mild traumatic brain injuries (mTBI) [114,115,116]. Postulations have been made from static balance impairments that dynamic balance control may be impaired and place those with prior mTBI at greater falls risk [116]. The postural performances may vary among populations, such as military versus civilian, and thus, requires normative data or baseline values to be used for comparisons to “normal” values [117]. However, the ability to assess balance in multiple varieties shows the breadth of applicability of force plate testing in military populations.

**Table 3:** List of Movements their associated Reliable Metrics, and Tactical Relevance.

Movement	Reliable Metrics	Tactical Relevance
Countermovement Jump Drop Jump Depth Jump	Jump height Modified reactive strength index Scaled mean propulsive power (power/body mass)	Explosive neuromuscular performance: ○ Speed, agility, power ○ Breaching, jumping over obstacles ○ Combat movement techniques
Countermovement Jump Drop Jump Depth Jump	Countermovement depth Braking/eccentric impulse Contraction time Propulsive/concentric rate of force development Propulsive/concentric impulse Propulsive/concentric peak force	Used for explaining indications of chronic fatigue as noted by performance reductions (i.e., pre- to post- training cycle or deployment) More sensitive for detecting acute neuromuscular fatigue (i.e., pre- to post- long-range patrol, highly demanding weekly training loads)
Drop Jump Depth Jump	Landing/eccentric peak force Landing / eccentric impulse Landing / eccentric rate of force development	Ability to absorb/ reduce impact forces Ability to land properly from elevated terrain or vehicles and traverse over rugged landscape
Loaded Countermovement or Drop Jumps	Jump height Modified reactive strength index Scaled mean propulsive power (power/body mass)	Explosive neuromuscular performance capabilities under loaded conditions Afford the greatest ecological validity with movement demands in tactical populations.
Loaded Drop Jumps	Landing/eccentric peak force Landing/eccentric impulse Landing/eccentric rate of force development	Ability to absorb/ reduce impact forces under load Ability to land properly from elevated terrain or vehicles and traverse over rugged landscape while equipped Improved ecological validity with demands of tactical populations
Isometric Mid-thigh Pull	Peak force Instantaneous forces	Maximal lower body strength Related with ability to lift equipment or personnel
Isometric Push to Pull Ratio	Peak force Impulse over 200 ms	Maximal upper body strength balance Related with ability to manipulate environment: ○ Breaching doors in forced entry ○ Handle-operated equipment ○ Shoulder injury risks
Plyometric Pushups Punching Variations	Peak force Mean propulsive power.	Explosive neuromuscular performance capabilities of upper extremities: ○ Hand-to-hand combat ○ Operating/loading equipment ○ Prone and vertical movements (e.g., bounding rush, rope climbing)

## 7. Conclusions

In the military, it is necessary to ensure progressive overloading is properly managed, which we now know is possible through controlled, objective monitoring of training loads and physical capacities, to improve performances and reduce the risk of injury. Due to the information provided in this review, it is recommended that force plate testing become a routine part of standard operating procedures in the military for NP monitoring and additionally for both acute and chronic NMF. Practitioners will then be able to engage in evidence-based practices to more effectively prescribe training loads over time so that physical characteristics such as explosiveness, maximal strength, and endurance are at the very least maintained, if not ideally enhanced, over the course of military personnel’s tenure. However, the complexity of force plate testing comes with required necessary precautions to ensure the monitoring procedures in place are reliable and faithful enough to make sound decisions. In conclusion, even though force plate testing may be a beneficial objective tool for monitoring, there is still a subjective nature to the quality of data that relies on solid preparation of the testing protocols, coaching and cueing to confirm maximal efforts, and selection of reliable and practical metrics that can be easily portrayed.

## Figures and Tables

**Table 1:** Summary of Force Plate Equipment, Movement, and Data Best Practices for NP Monitoring.

**Equipment**	Regular force plate calibration Use the same or similar equipment each time (i.e., PVC pipe for no arm swing jumps, weight vest or barbell for loaded jumps) Force plate conditions are consistent for every test (i.e., type and level flooring) Consistent mechanics (i.e., knee and hip angle in the isometric mid-thigh pull) Thresholds used to determine key moments of the movement If loaded, ensure bodyweight includes external load Choose valid equipment, consider system specifications when comparing across devices (e.g., strain gauge vs. piezoelectric, filtering, integration techniques, and sampling rates)
**Movement**	Ensure the individual performs at maximal effort Consistent cues: “Upon the ‘Go’ command, jump as high and as explosively as possible” Consistent instructions are provided for each movement (i.e., drop jump versus depth jump/jump with minimum ground contact versus jump as high as possible upon ground contact no matter what means necessary) Use a short, standardized warm-up prior to conducting the test Determine right movement for population and keep consistent (squat jump, arm swing, and etc.) Take care in initial bodyweight measurement; remain still during the weighing phase for at least one second prior to testing (essential to identify thresholds for movement onset and derivation of acceleration-time curves) Practitioner administering the test is trained and well versed in all Equipment, Movement, and Data best practices Tactical personnel is familiar and comfortable with the movement
**Data**	Rely on reliable metrics for each test Use testing metrics that are proven to be related with performances or theoretically relevant to the occupational specialties of the individual Conduct regular internal reliability checks to confirm reliability of measurements When possible, run multiple (2–3) trials and use the mean of all In case of an improper trial, which can be assessed by eye, remove trial to avoid averaging an inaccurate trial Scaling metrics, relative to an individual’s body mass, can help to adjust the force-time metrics to control for body mass when making comparisons between individuals or over time Compare Individuals to themselves over time (neuromuscular fatigue) Compare Individuals to groups (profile testing)
